# Just one look: Direct gaze briefly disrupts visual working memory

**DOI:** 10.3758/s13423-016-1097-3

**Published:** 2016-06-29

**Authors:** J. Jessica Wang, Ian A. Apperly

**Affiliations:** 1 School of Psychology, University of Birmingham, Edgbaston, Birmingham, B15 2TT UK; 2 School of Psychology, Keele University, Keele, ST5 5BG UK

**Keywords:** Direct gaze, Eye contact, Visual working memory, Social cognition

## Abstract

Direct gaze is a salient social cue that affords rapid detection. A body of research suggests that direct gaze enhances performance on memory tasks (e.g., Hood, Macrae, Cole-Davies, & Dias, *Developmental Science*, *1*, 67–71, 2003). Nonetheless, other studies highlight the disruptive effect direct gaze has on concurrent cognitive processes (e.g., Conty, Gimmig, Belletier, George, & Huguet, *Cognition*, *115*(1), 133–139, 2010). This discrepancy raises questions about the effects direct gaze may have on concurrent memory tasks. We addressed this topic by employing a change detection paradigm, where participants retained information about the color of small sets of agents. Experiment 1 revealed that, despite the irrelevance of the agents’ eye gaze to the memory task at hand, participants were worse at detecting changes when the agents looked directly at them compared to when the agents looked away. Experiment 2 showed that the disruptive effect was relatively short-lived. Prolonged presentation of direct gaze led to recovery from the initial disruption, rather than a sustained disruption on change detection performance. The present study provides the first evidence that direct gaze impairs visual working memory with a rapidly-developing yet short-lived effect even when there is no need to attend to agents’ gaze.

## Introduction

To successfully navigate the social world, it is fundamental for individuals to be able to detect and understand sociocommunicative signals, such as eye gaze (Kleinke, 1986). Eye gaze triggers reflexive orientating of an observer’s attention (e.g., Friesen & Kingstone, 1998), reveals information about mental states (Baron-Cohen, 1995), and direct gaze (also known as eye contact) can signal an intention to communicate with others (Csibra & Gergely, 2009). The ability to detect direct gaze is evident within the first few days after birth (Farroni, Csibra, Simion, & Johnson, 2002). The early sensitivity to direct gaze suggests that it is likely to be a key building block for the development of social skills. A number of studies demonstrated that direct gaze facilitates face-related processes, social fluency, and memory for speech (Adams, Pauker, & Weisbuch, 2010; Conty & Grèzes, 2011; Fullwood & Doherty-Sneddon, 2006; Hood, Macrae, Cole-Davies, & Dias, 2003; Senju, Hasegawa, & Tojo, 2005). Nonetheless, other studies have found that maintaining eye contact or merely observing direct gaze hinders performance on cognitive tasks (Conty, Gimmig, Belletier, George, & Huguet, 2010; Markson & Paterson, 2009; Riby, Doherty-Sneddon, & Whittle, 2012). The present study assesses whether direct gaze has a facilitative or disruptive effect on visual working memory. We also discuss the context in which direct gaze may facilitate and disrupt task performance.

### Direct gaze facilitates sociocommunication and social fluency

Visual attention studies indicate that adults (Conty, Tijus, Hugueville, Coelho, & George, 2006) and typically developing children (Senju et al., 2005) are quicker to detect direct gaze stimuli compared to averted gaze stimuli in visual search tasks. Interestingly, Senju et al. (2005) demonstrated that, when the face stimuli were inverted, both adults and typically developing children showed a reduced search advantage for direct gaze stimuli, suggesting that the beneficial effect of direct gaze depends upon processing faces as a social stimulus. Face–memory studies suggest that direct gaze facilitates face recognition both when the faces were encoded deliberately (Hood et al., 2003) and when there was no requirement to encode faces (Conty & Grèzes, 2011). Adams et al. (2010) revealed that the cross-race memory effect, which is the relatively poor other-race-face recognition compared to own-race-face recognition, only occurred when the faces displayed direct gaze and not when the faces displayed averted gaze. These studies indicate that direct gaze not only plays an essential role in face-related processing but it can also modulate the way in which social signals are perceived and interpreted.

### Direct gaze disrupts cognitive processes

In contrast to the studies of social processing described above, a number of findings suggest that direct gaze plays a disruptive role in cognitive tasks. Conty et al. (2010) showed that, when individuals performed a Stroop task, the presence of a pair of isolated irrelevant eye stimuli displaying direct gaze led to an exaggerated Stroop interference effect. However, individuals’ performance was unaffected when the irrelevant eye stimulus displayed averted gaze or when the eyes were closed. A similar disruptive effect of direct gaze was also observed in studies that require participants to maintain eye contact with a real person while completing a matrix task (Markson & Paterson, 2009) or a mathematical task (Riby et al., 2012). Taken all together, these findings suggest that the state of “being looked at”— either by a person or by seeing a direct gaze stimulus—hinders concurrent performance on tasks that require cognitive control. This pattern is in tension with the studies described earlier that highlight the facilitative effect direct gaze has upon social perceptual tasks.

While both the facilitative and disruptive effects of direct gaze have been documented in cognitive tasks, presently little is known about the effect direct gaze may have on memory for stimuli other than faces, which may be a special case (e.g., Adams et al., 2010; Hood et al., 2003). We focus here on visual working memory because of the critical role that it is commonly held to play in bridging between perceptual inputs and the formation of conceptual representations (Jiang, Makovski, & Shim, 2009). Social cognition often requires rapid integration of perceptual inputs—such as the bodily movements and direction of gaze—and therefore it might be expected to make significant demands on visual working memory. It is also the case that social cues such as agents’ actions (Wood, 2007), faces (e.g., Scolari, Vogel, & Awh, 2008) and eye gaze (Doherty-Sneddon, Bonner, & Bruce, 2001) are stimuli we frequently encounter and are known to attract visual attention. This leads to the possibility for social stimuli to dominate processing at the expense of other information. However, the visual working memory literature has a primary focus on the encoding and retrieval of non-social information in non-social contexts (Hyun, Woodman, Vogel, Hollingworth, & Luck, 2009; Luck & Vogel, 1997), and while other studies have examined the effect of enforced gaze on other aspects of memory performance, no study has examined the incidental effect of task-irrelevant direct gaze on visual working memory. We conducted two experiments to do so. Rather than requiring participants to sustain mutual gaze (e.g., Doherty-Sneddon et al., 2001), the direct gaze stimuli were brief and incidental to participants’ main task, as they often might be in real social contexts. We varied the timing of stimulus presentation in two experiments in order to understand the timecourse of possible effects of direct gaze on visual working memory processing.

## Experiments 1a and 1b

### Experiment 1a

The current experiment compared change detection performance for displays in which agents directly look towards participants to displays in which agents look towards objects. Participants’ task was to detect changes amongst the colors of the agents and the shapes of the objects. The agents were not differentiated by their eye-features, therefore processing the agents’ eye gaze was not advantageous for task performance. The current design had the direct gaze stimuli embedded in the agent stimuli, as this was a somewhat more naturalistic presentation of direct gaze compared to having a pair of eyes singled out from the context of a face (see Conty et al., 2010).

#### Method

##### Participants

Sixteen students (14 females, mean age 20.06 years, age range 18–23 years) from the University of Birmingham took part in this experiment in return for a small honorarium or course credits. All participants had normal color vision and normal or corrected-to-normal visual acuity. All participants’ change detection accuracies were within two standard deviations from the means in all four conditions, therefore no participants’ data were excluded from the analysis.

##### Design and procedure

**Fig. 1:**
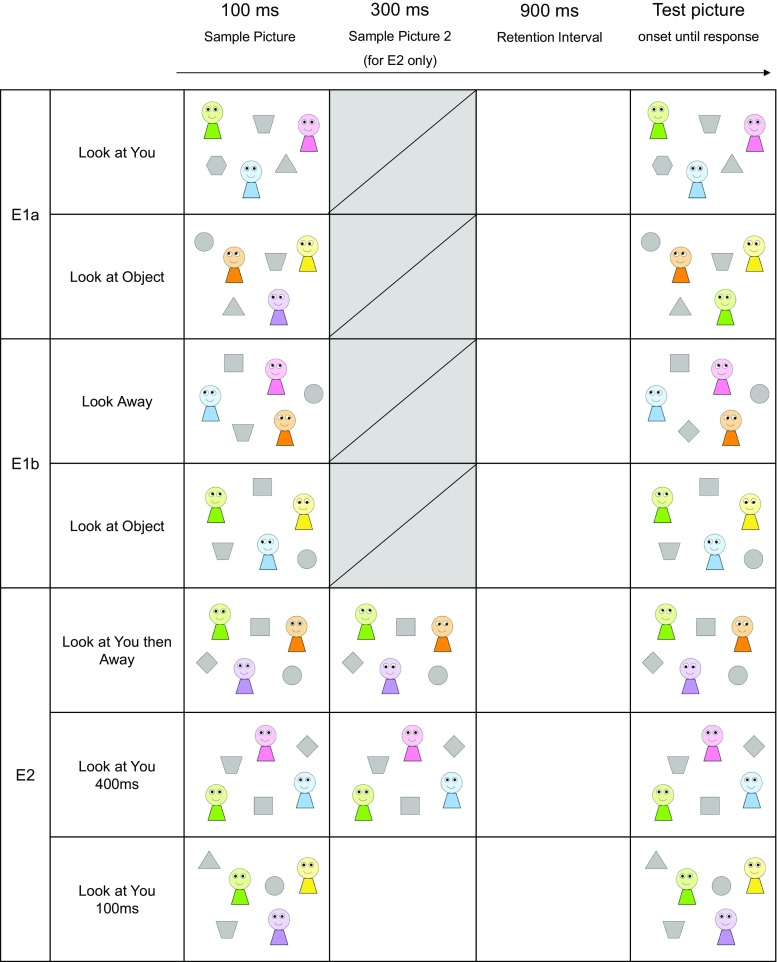
Examples of trial sequences from Experiments 1a, 1b, and 2. Sequences began with the displays on the *left* and progressed towards to the *right*. Experiments 1a and 1b included blocks in which either an agent or object could change. In Experiment 2, only the agent could change. Displays in this figure are selected examples from the full set, to illustrate the different trial types

A 2 × 2 within subject design was constructed with gaze direction (look-at-you, look-at-object) and change element (agent-change, object-change) as factors. Each display contained either 3 or 4 agents along with a matching number of objects.^1^ There were 25 different displays for each set size. These displays were generated from a pool of 6 agents of different colors (yellow, violet, green, pink, blue, and orange) and 6 objects of different shapes (circle, triangle, square, diamond, trapezoid, and hexagon). No two agents in a given display were the same color, and no two objects were the same shape. In the look-at-object condition, each agent always looked towards an object. In the look-at-you condition, the agents always looked straight ahead giving the impression that they were looking towards the participants. A one-shot change detection paradigm was employed (e.g., Luck & Vogel, 1997, see also Rensink, 2002 for a review). Each sample picture was presented for 100 ms, followed by a 900-ms retention interval, and finally a test picture was displayed until participants made a response (see Fig. 1). Participants were instructed to left-click on the computer mouse when they saw a change in the test picture from the sample picture, and right-click when they saw no change.

All displays in the current study subtended 7.3° × 9.8° in the center of a computer screen. The objects subtended 1.34° × 1.34° on average. The agents each subtended 2.38° × 1.43°, their color-coded body subtended 1.05° × 1.24°. Half of the time, the test pictures were identical to the sample pictures, the other half of the time, the test pictures contained a change from the sample pictures. The change element was in either the shape of one of the objects or the color of one of the agents. The two types of changes occurred equally frequent. The agent-change trials and the object-change trials were presented in separate blocks, therefore participants were able to anticipate changes to occur either amongst the colors of the agents or amongst the shapes of the objects. A total number of 384 test trials were presented in four test blocks of 96 trials. Each test block was preceded by four additional practice trials from the same condition. The experiment was presented with E-prime (Schneider, Eschman, & Zuccolotto, 2002).

##### Predictions

If direct gaze facilitates information encoding, then the level of change detection accuracy in the look-at-you condition should be higher than the look-at-object condition. However, if direct gaze disrupts visual working memory, then participants should show lower level of accuracy in the look-at-you condition compared to the look-at-object condition (see Fig. 1). It is important to note that participants had just 100 ms to encode the color of the agents or the shape of the objects, giving participants a strong incentive to ignore any irrelevant element of the display in order to optimize performance.

#### Results and discussion

**Fig. 2:**
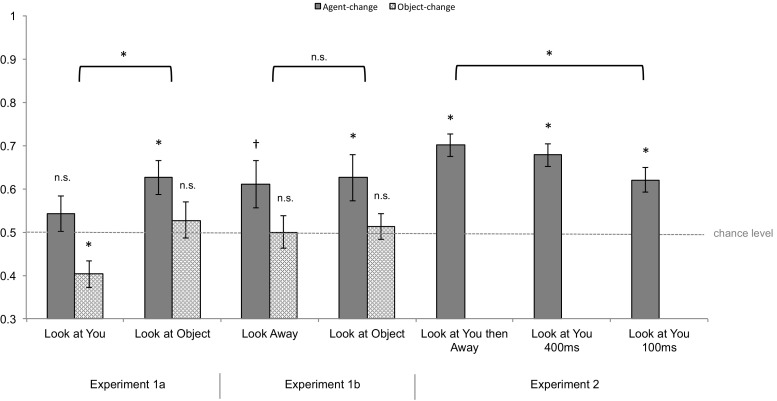
Proportion correct for the agent-change conditions from all experiments. *Error bars* represent one standard error from each condition’s mean. The *asterisks* mark the conditions that were performed at levels significantly different from chance (individual *bars* either *above* or *below* chance) or showed a significant main effect of gaze direction (*square brackets*). The *dagger* marks the condition that was marginally significantly above chance level

The proportion of correct responses on change trials for each condition was computed,^2^^.^ 3 Preliminary analysis was conducted to test conditions against the proportion correct predicted by chance level of .50 (see Fig. 2). As shown in Table 1, the current results revealed that participants detected changes in displays containing agents looking directly at them less accurately compared to agents looking at objects. The poor performance in the look-at-you condition is consistent with the outcome predicted by a disruptive effect of direct gaze. This effect can be interpreted with confidence for judgments about agents, which were made at above-chance levels. More caution is necessary for interpreting judgments about objects, which were not performed above chance level. Nonetheless, the absence of an interaction effect between gaze direction and change element (see Table 1) indicates that the disruptive effect of direct gaze likely generalize over memory for objects and agents.

**Table 1:** (a) Results from repeated-measures ANOVAs in Experiments 1a, 1b, and 2. (b) Paired comparisons following a significant main effect of gaze direction in Experiment 2. (c) Independent *t* test comparing performance levels following 100 ms of direct gaze and 900 ms versus 1200 ms of retention interval. All *t* tests were two-tailed

(a) ANOVA					
		*F*	*df*	*p*	*ηp* ^2^
E1a	Gaze direction (look-at-you, look-at-object)	29.52	15	<.0001	.663
	Change element (agent-change, object-change)	9.19	15	.008	.380
	Gaze direction × Change element	0.64	15	.437	.041
E1b	Gaze direction (look-away, look-at-object)	0.45	14	.514	.031
	Change element (agent-change, object-change)	3.03	14	.103	.178
	Gaze direction × Change element	0.02	14	.888	.001
E2	Condition (look-at-you-then-away, look-at-you-400 ms, look-at-you-100 ms)	6.91	60	.002	.188
(b) Post hoc *t* test					
		*t*	*df*	*p*	*d*
E2	look-at-you-then-away vs. look-at-you-400 ms	−1.21	30	.235	-
	look-at-you-then-away vs. look-at-you-100 ms	3.27	30	.003	0.53
	look-at-you-400 ms vs. look-at-you-100 ms	−2.50	30	.018	−0.38
(c) Independent *t* test					
		*t*	*df*	*p*	*d*
E1a vs. E2	E1a look-at-you (100 ms) vs. E2 look-at-you-100 ms	−1.60	45	.118	-

While it is possible that direct gaze disrupted encoding, it is also possible that the agents’ object-oriented gaze led to more efficient information encoding. This account receives indirect support from findings that in a crowded natural scene, the closer an object was located to the direction of an agent’s uninformative gaze, the quicker it was detected (Langton, O’Donnell, Riby, & Ballantyne, 2006). If such an effect resulted in better encoding of the entire scene (including the agents themselves), then this might explain superior performance in the look-at-object condition of the present experiment. Experiment 1b was designed to distinguish between the potential effects of direct gaze and object-oriented gaze observed in Experiment 1a. This design excluded any direct gaze component, replacing the look-at-you condition with a look-away condition (see Fig. 1). Both conditions in Experiment 1b contained agents displaying averted gaze, therefore it affords a close comparison between object-oriented gaze and non-object-oriented gaze. If the agents’ object-oriented gaze led to more efficient information encoding in Experiment 1a, then a higher level of change detection accuracy should be observed in the look-at-object condition compared to the look-away condition in Experiment 1b. However, if the observed difference between the look-at-object and look-at-you conditions was a consequence of a disruptive effect of direct gaze, then no differences should be found between the two averted gaze conditions in Experiment 1b.

### Experiment 1b

#### Method

##### Participants

Fifteen students (13 females, mean age 18.93 years, age range 18–23 years) from the University of Birmingham took part in this experiment in return for course credits.

##### Design and procedure

A 2 × 2 within-subject design was constructed with gaze direction (look-away, look-at-object) and change element (agent-change, object-change) as factors. The rest of the design and procedure was identical to that of Experiment 1a.

##### Results and discussion

As shown in Table 1 and Fig. 2, participants performed at similar levels of accuracies in the two averted gaze conditions. This indicates that the agents’ object-oriented gaze does not lead to improved change detection. Therefore, the difference between the look-at-object condition and the look-at-you condition in Experiment 1a was likely to be caused by a disruptive effect of direct gaze. In a further experiment, we investigated the nature of this effect.

## Experiment 2

Clearly, any disruption to visual working memory from direct gaze can only be temporary, therefore Experiment 2 was designed to examine the conditions necessary to recover from the disruption caused by direct gaze. After an initial period of direct gaze, we presented three types of stimuli: averted gaze, continued direct gaze, or a blank screen (see Fig. 1). As such, we hypothesized two main possible routes to recovery^4^: the first possibility was informed by studies of attentional cueing (see Frischen, Bayliss, & Tipper, 2007 for a review). These studies mostly employed trial sequences that included direct gaze stimuli rapidly followed by the same face with an averted gaze, giving the compelling impression of a gaze shift. This sequence leads to reflexive orientation of attention to the location gazed at by others. Hence, presenting averted gaze stimuli after the initial direct gaze stimulus may lead the direct gaze to be reinterpreted, perhaps attenuating its disruptive effect on visual working memory. A classic study by Driver et al. (1999) demonstrated that presenting averted gaze for 300 ms, but not 100 ms, prior to the onset of a target in a localization task provided sufficient time for the direction of eye gaze to be processed and produce a reliable gaze-cueing effect. Therefore, in the present experiment, we presented averted gaze stimuli for 300 ms following the initial 100 ms of direct gaze to ensure that the direction of eye gaze can be processed (the look-at-you-then-away condition). This condition was employed to signpost the level of change detection performance following a recovery from the disruptive effect of direct gaze. Secondly, we hypothesized that reinterpretation of direct gaze might not be necessary. Instead, recovery may occur if participants were simply given time to encode the stimuli after any initial disruption caused by the onset of a direct gaze stimulus. We compared trial sequences in which the initial 100 ms of direct gaze stimulus was extended for a further 300 ms before the blank screen retention interval (giving time for encoding after spontaneous recovery from initial disruption), with trial sequences where the 100 ms of direct gaze stimulus was followed by 300 ms of a blank screen before the retention interval (giving no further encoding opportunity, but matching the overall length of the trial sequence).

### Method

#### Participants

Thirty-one students (25 females, mean age 19.78 years, age range 18–20 years) from the University of Birmingham took part in this experiment in return for course credits. An additional participant’s data were excluded from the analysis due to having change detection accuracies two standard deviations below the mean in two of the three conditions.

#### Design and procedure

Three conditions were included in a within subject design (look-at-you-then-away, look-at-you-400 ms, look-at-you-100 ms). Since performance in the object-change conditions in Experiment 1 was consistently at or below chance level, here we excluded those trials from the design entirely, so participants always detected color changes in the agents. In the current experiment, all trials began with 100 ms of direct gaze display, which was identical to the sample pictures from Experiment 1a. This display was followed by a 300-ms display in which the agents looked away, continued to look at the participants, or the screen turned blank for the same duration. When the agents looked away, participants only saw the agents’ gaze directions shift from direct gaze to averted gaze; the rest of the screen remained identical across the two displays. This was followed by 900 ms of retention interval before a test picture onset until a response was detected (see Fig. 1). A total number of 288 test trials were presented in three test blocks of 96 trials. Each test block was preceded by four additional practice trials from the same condition. The remainder of the design and procedure was identical to Experiment 1a.

### Results and discussion

As shown in Table 1 and Fig. 2, compared with the baseline in which 100 ms of a direct gaze stimulus was followed by a blank screen, performance improved when this initial direct gaze stimulus was either extended by a further 300 ms or followed by 300 ms of averted gaze. The fact that improvement was observed regardless of whether the extended exposure to the stimuli included direct or averted gaze suggests that the critical factor for recovery is the additional encoding opportunity. This additional 300 ms of encoding time allowed participants to overcome the initial disruption from direct gaze, and subsequently encode the identities of the agents. These findings suggest that direct gaze causes a rapid (within 100 ms) yet short-lived (shorter than 400 ms) disruption on change detection performance. Interestingly, change detection performance did not differ following a 900-ms (Experiment 1a look-at-you) versus 1200-ms (Experiment 2 look-at-you-100 ms) retention interval, suggesting that the disruption likely occurred during encoding, not maintenance.

## General discussion

The present study provides the first evidence that direct gaze impairs visual working memory with a rapid yet short-lived effect, even when there is no need to attend to agents’ gaze. Experiments 1a and 2 revealed that change-detection accuracies were low when briefly presented displays contained agents directly gazing towards participants. All three experiments provided clear evidence for above-chance change-detection accuracies for briefly presented displays that did not contain agents gazing directly at participants. Furthermore, the recovered change detection performance in the look-at-you-400 ms condition of Experiment 2 suggests that direct gaze had its disruptive effect during encoding, not retrieval. The present finding implies that, while visual working memory would normally piece together all the snapshots we obtain through saccades and fixations, direct gaze can cause a temporary disruption in this system by compromising the quality of the early snapshots.

The present findings that direct gaze impairs visual working memory appear to be in tension with the findings of Adams et al. (2010), Conty and Grèzes (2011), and Hood et al. (2003). All three studies showed higher levels of accuracies in face recognition when faces were seen displaying direct gaze compared to averted gaze. In contrast, the current findings showed poorer performance on memory task when the agents displayed uninformative direct gaze rather than averted gaze. There are two noteworthy distinctions between the current study and the studies described above. Firstly, the presentation duration of direct gaze in the three face memory studies were considerably longer (between 1300 ms and 5000 ms) compared to the 100 ms presentation in the current study. As demonstrated in Experiment 2 of the current study, it is likely that when direct gaze was seen for more than 100 ms, participants were able to strategically overcome the initial disruption of direct gaze with enough time in hand to encode the remaining information. Secondly, in these three studies participants had to recognise faces in which agents’ eyes were likely to provide useful information. In contrast, in the current study, the information necessary for detecting a change of agent came from the color of the agents and the shapes around them, not from their eyes. The irrelevance of direct gaze in the current study bears similarity to Conty et al. (2010), where direct gaze stimuli caused compromised performance on a concurrent Stroop task. It is likely that the way in which direct gaze attracts attention (Conty et al., 2006; Senju et al., 2005) drives the effects in different directions, depending on the task at hand. It follows that direct gaze facilitates tasks where eye gaze is informative, such as those involving processing of facial and emotional contents, but it hinders performance on tasks that do not require participants to process eye gaze, especially over short time intervals. These effects are important for understanding memory and attention in rapidly moving social contexts because they suggest that direct eye gaze will help or hinder judgments depending on whether the information they carry is relevant or irrelevant to the task, and on whether the situation allows time for recovery from the potentially disruptive effects of direct gaze. Future research should focus on the timecourse in which direct gaze positively or negatively affect memory for social versus non-social information.

## Footnotes

1 The set size was originally included as a factor. However, both set sizes yielded highly similar results across all experiments of this study (all interaction effects involving set size *F*s < 0.23, *p*s > .640). Henceforth, the two set sizes were collapsed in the descriptions of the design and analyses.

2 The same patterns of results were observed when the analyses were carried out on d-prime scores instead of the proportion of correct responses in all experiments.

3 As is common in similar change-detection paradigms (e.g., Luck & Vogel, 1997), our task was designed to test participants’ accuracy, and participants were not put under any time pressure. This makes speed–accuracy trade-offs unlikely. Nonetheless, to address a reviewer’s concern, we extracted the RT data and showed that there was no sign of any speed and accuracy trade-off. Furthermore, RTs on change- and no-change-trials were highly similar.

4 Note that ‘recovery’ in this context is not defined by 100 % change-detection accuracy. Instead, it is the level of change-detection performance in the absence of a disruptive effect of direct gaze.
